# Progress in Surgery

**Published:** 1897-05-22

**Authors:** 


### Progress in Surgery.

### BRAIN SURGERY.

**Diagnosis of Cerebral Abscess.**—Eskridge,[1] in concluding an elaborate paper on the diagnosis of chronic abscess of the brain, states that although in many cases an absolute diagnosis is impossible, this is not to deter the surgeon from giving the patient the chance which an operation promises. "The physician who has not the courage to recommend an exploratory operation in a strongly probable case of abscess of the brain lest he may be wrong in his diagnosis, is more solicitous for his own reputation than for the welfare of his patient." A careful study of each individual symptom and of the apparent etiological factors in any case is most important, although these are sometimes misleading. In one case reported by Eskridge, chronic otorrhœa, a blow to the head and suppuration in a distal part of the body were noted, and yet the patient died of cyst and tumour of the brain. In another, arterial thrombosis was strongly suggested by the symptoms, yet the patient died of intracranial suppuration. In another the symptoms and apparent etiology pointed to tuberculous meningitis, although the actual condition was suppuration. The differential diagnosis of chronic brain abscess is from tuberculous meningitis, cerebral softening, hæmorrhage, and tumour: (1) In tuberculous meningitis the majority of cases run their course in a few weeks; there is fever, and symptoms of involvement of cranial nerves are usually present. Unfortunately these symptoms vary greatly. (2) Cerebral softening and focal encephalitis is not often a source of difficulty in diagnosis, but Eskridge, and also Murri, have both met with cases. The writer can give no points of definite value in the differential diagnosis. (3) Cerebral hæmorrhage, coming on suddenly and bursting into the ventricles in one case of the author's, was mistaken for an abscess rupturing in the ventricle, but this must be rare. (4) Tumour. This is, perhaps, the most difficult diagnosis of all. The history of a possible cause and the existence of fever point to abscess, but not certainly, because sub-normal temperature is very common, and injuries and middle-ear suppurations often occur in the presence of tumours. The degree of optic neuritis present in relation to the existence of the disease may aid one. A steady, but gradual, increase of symptoms of organic disease of the brain, with the development of one nerve root symptom after another, is in favour of tumour, so are prolonged intervals during which the symptoms make but little progress.

Oppenheim[2] has also studied the differential diagnosis of cerebral abscess. We have already referred to his conclusions regarding abscess the result of trauma. Respecting the diagnosis of abscess following on otitis, he points out that there are two distinct groups of conditions from which it has to be excluded: (1) Other cerebral affections due to ear disease, such as extra-dural abscess, sinus thrombosis, meningitis, &c.; and (2) cerebral conditions independent of ear mischief, such as tumour, encephalitis hæmorrhagica, tuberculous, and other forms of meningitis, and so on. The author agrees with most observers in stating that in cerebral abscess the temperature is sub-normal and the pulse slow; but in some cases slight and temporary elevation of temperature is met with. There are cases in which certain symptoms usually associated with cerebral abscess are present, such as headache, vertigo, vomiting, and slight stupor in the presence of a septic ear condition, and yet on operating no abscess is found and the patient recovers. It seems impossible to avoid this error. The author is careful to emphasise the point that the presence of hysterical manifestations must not be allowed to weigh too heavily against a diagnosis of abscess. The author describes 35 cases of his own in which there was reason to suspect cerebral abscess. In 14 of these abscess was correctly diagnosed, in 18 it was correctly excluded, and in three it was wrongly diagnosed, the conditions present being purulent meningitis, tuberculous meningitis, and uncomplicated otitis.

A case of some interest is reported by Gorham Bacon,[3] in which a young man of 32, after having suffered from chronic suppuration of the middle ear on the left side, began to suffer from pain in the head, with high temperatures (101 deg. to 104 deg. F.), occasional rigors, rapid pulse (104), and general convulsions followed by unconsciousness. The mastoid was explored, and in the process the lateral sinus and facial nerve were both injured. As the general symptoms were not relieved, the temporo-sphenoidal lobe was exposed by trephining, and a large abscess evacuated. Recovery took place. In this case the symptoms were those of sinus thrombosis rather than of cerebral abscess.

In a recent discussion, Allen Starr stated that he had seen 12 cases of brain abscess operated upon. In other two cases the diagnosis of abscess was wrong, thrombosis and general meningitis being the conditions present. Three of the cases operated upon recovered. This observer lays less stress on the low temperature and slow pulse in cerebral abscess than Macewen does. Sub-cortical aphasia is a frequent symptom in left-sided cerebral abscess.

**Otogenous Pyæmia.**—In a recent work by Hessler,[4] much stress is laid on the importance of prophylactic treatment, by careful management of early ear mischief, and the teaching of the modern British writers is amply corroborated. W. R. H. Stewart[5] reports a recovery after clearing out a clot from the lateral sinus, associated with middle-ear disease. The temporo-sphenoidal lobe and cerebellum were explored with negative results. J. K. Love,[6] of Glasgow, reports another.

**Operations on the Mastoid.**—Bronner, of Bradford, gives a *résumé* of the various operations which have from time to time been performed for suppuration in the mastoid process, emphasising the value of prompt and thorough measures. He prefers Macewen's modification of Stacke's operation, the pulling down of the ear giving better access. The only difficulties in the procedure are due to anatomical variations in the position of the antrum, lateral sinus, facial canal, and floor of the middle fossa. To keep the large cavity formed open, a flap is taken from the posterior part of the auricle and stitched to the skin or periosteum behind. In some cases Schwartze makes a permanent opening behind the meatus and ear. The indications for opening the mastoid are given as follows: (1) Acute primary or secondary inflammation of the mastoid process, when symptoms do not improve after a few days' treatment. (2) Chronic inflammation of the mastoid with recurrent attacks of swelling. (3) Fistula over or near the mastoid. (4) Chronic inflammation of middle ear without apparent affection of mastoid, if there are symptoms of retention of pus, diseased bone, or cholesteatoma. (5) Persistent pains over the mastoid. (6) Chronic otorrhœa, with evidence that inflammation has spread beyond the middle ear.

Love, of Glasgow,[8] calls attention to a simple and safe method of opening the mastoid antrum. The gouge and mallet are associated with danger, and the burr is slow. He uses a set of ordinary joiner's brogs having projecting shoulders at intervals of an eighth of an inch, to prevent too sudden entry with risk to the interior. If the shortest brog does not reach the cavity the next is tried, and so on until the antrum is reached. The opening is then enlarged with the burr or gouge.

[1] Amer. Jour. Med. Sciences, Sept., 1896. [2] Berliner Klin. Wochen., No. 45, 1896. [3] New York Med. Jour., Aug., 1896. [4] Die Otogene Pyämie, Jena, 1896. [5] Lancet, Nov., 1896. [6] Glasgow Med. Journ., Sept., 1896. [7] Brit. Med. Jour., Oct., 1896. [8] Glasg. Med. Jour., Sept., 1896.

### BRAIN TUMOURS.

**Some Points in Diagnosis.**—The localisation of brain tumours for operative purposes is often a matter of extreme difficulty. Sinkler[1] points out that there are many cases in which tumours of the mid-brain give rise to symptoms which closely resemble those of a growth in the motor area. Growths which are most likely to give rise to errors in diagnosis are those which begin in a "silent region" like the frontal lobe, and which gradually encroach upon the motor area. Two illustrative cases are reported. (1) A man, aged 25, had a general convulsive seizure without any assignable cause. Nine months later he had another, and after that had many at varying intervals. The clinical symptoms pointed to a lesion (probably neoplastic) in the region of the face centre of the right side, but on operating no such tumour was found. Temporary relief followed the operation, but symptoms returned and a second operation was performed at the same place. This time a well-developed tumour presented itself, but on account of severe bleeding could not be removed. The patient died, but no autopsy was made. (2) A man healthy till 50 years of age, when he suddenly and unaccountably had convulsive seizures affecting the right side alone. The symptoms and progress of the disease pointed to a cortical lesion, at first irritative, and later becoming destructive. On operating no tumour was found, but after death the frontal lobe was discovered to be the seat of a new growth, which to the naked eye did not appear to involve the motor region directly. A writer in the *American Medico-Surgical Bulletin* of December, 1896, draws attention to the similarity of symptoms which exists in certain cases between tumours of the cerebellum and of the frontal lobe, anterior to the motor area. As example, a case is referred to where the pain was frontal, right-sided, with loss of smell which began on the right side, but the mental power was intact. Typical cerebellar symptoms were absent, yet on post-mortem examination the tumour was found in the cerebellum. Williamson's paper[2] is quoted, and its general conclusions summarised. Fifty cases of focal disease in the prefrontal region were critically analysed, and the following facts came out: Region—Left side more frequent than right; bilateral in 20 per cent.; in 30 per cent. lesion was cortical, in 20 per cent. basal. Headache entirely frontal in 44 per cent.; in 36 per cent. of cases in which headache was localised it was occipital, with or without frontal pain. Tenderness on percussion of skull present in 75 per cent., and in most of the cases the growth was near the tender area. Optic neuritis present and bilateral in 64 per cent., absent in 10 per cent. Smell was affected in 41 per cent. Paralysis very slight and late in illness, due to extension back to motor area. Convulsions occurred frequently. Anæsthesia never recorded. Ataxia not mentioned in 66 per cent. Unsteadiness and reeling present in 28 per cent. Knee-jerks absent in 20 per cent., normal or increased in 53 per cent., feeble on both sides 7 per cent., feeble on side opposite tumour 13½ per cent., feeble on side of tumour 7 per cent. Mental symptoms—In 71 per cent. there was mental decadence, a dull mental state, loss of power of attention, loss of memory and of spontaneity, sleeplessness or semi-comatose condition. In a few cases patients were excitable and suspicious. This table shows the point on which most value may be laid for diagnostic purposes to be the mental state. Most of the other symptoms are common to cerebellar and prefrontal tumours moie or less.

**Tuberculoma of Brain.**—Kronlein[3] reports a case in which he successfully removed a conglomerate tubercle from the brain. Such results are rare because the disease is seldom primary in the brain, and even when it is the foci are multiple and diffused. The mortality is about 50 per cent.

Williamson[4] reports a case in which a tuberculous cerebral tumour became encapsulated, and unilateral convulsions which had been due to its presence ceased. Four years before being seen by Williamson the patient had suffered from left-sided Jacksonian epilepsy with impairment of vision. Eighteen months after the fits ceased. Some years later he suffered from ataxia, headache, vomiting, and double optic neuritis, as well as pulmonary phthisis. He died, and on post-mortem examination there was found an old encapsulated tuberculous nodule in the right cerebral hemisphere, a recent tuberculous mass in the cerebellum, and pulmonary tuberculosis.

Napier[5] also records the case of a girl aged 16, who suffered from left-sided headache, vomiting, and constipation, slight squinting, and later paralysis of left external rectus and dilated pupils (especially the left). There was evidence of old standing strumous keratitis, and on examining the fundus double optic neuritis was present. Two months later most of the symptoms had disappeared, but the optic nerves atrophied. The opinion of the author is that the patient suffered from a small tuberculous growth, accompanied by a localised meningitis involving the ocular nerves.

**Gummata of Brain.**—Durante[6] reports the case of a man who had suffered from pains in the head which were at first relieved by anti-syphilitic treatment. The benefit, however, was only temporary, and later he showed a prominence of the left fronto-parietal region, with exophthalmos and amblyopia, diminished memory, melancholy, and hallucinations. An operation was performed at the seat of swelling, and three hard syphilitic gummata removed from the frontal lobe. Next day his vision returned, and his other symptoms soon disappeared. Six months after he had a Jacksonian fit. In another case the removal of a hard, fibrous, adherent body from the brain produced marked benefit to the moral and intellectual condition.

**Glio-sarcoma.**—A case of glio-sarcoma of the cerebellum is reported by J. H. Lloyd.[7] This writer states that statistics seem to show that the cerebellum is the most common seat of intracranial new growth, about 25 per cent. being in this position. They occur oftenest in children and young adults, and the commonest varieties are tuberculoma, glioma, and sarcoma. In the malignant varieties a history of trauma is usually forthcoming, but its etiological value is often doubtful. In the case recorded the patient sustained a severe injury to the occipital bone several years before the onset of symptoms of tumour. In addition there was a subsequent history of syphilis. Clinically the tumour appeared to be in an accessible situation, and operation promised hopeful results. A rapid change for the worse after operation had been decided upon led to the abandonment of the idea. The points of special interest in the case were: 1. A high degree of optic neuritis. Blindness was one of the earliest symptoms. 2. There was almost complete ophthalmoplegia interna, but no ophthalmoplegia externa. 3. None of the cranial nerves below the fifth, with the exception of the eighth, were affected, although the tumour from its position might have been expected to press upon these. 4. There were no variations in the knee-jerks. 5. There was no apparent association between the previous injury and the tumour. 6. Forced movements were prominent and were towards the side of the lesion. 7. In the light of the autopsy the operation would most likely have been fatal.

Thomas and Keen[8] give an elaborate and interesting report of the successful removal of a large glio-sarcoma from the left frontal region of a lad of 17. The main clinical features were right facial paralysis, a thickness of speech, protrusion of the left eyeball, greater optic neuritis in left eye, marked change in the mental characteristics and activity, hebetude and indisposition to move about, and decided instability of motion. The absence of Jacksonian spasms pointed to its being sub-cortical. Its slow progress, lack of variability in its symptoms, and absence of pain and tenderness on percussion indicated a hard sarcoma, rather than a glioma, and gave good reason for operating. Keen operated by raising a very large osteoplastic flap, and removed a tumour weighing 2½ oz. and measuring 3½ in. long, 2½ in. broad, and 1$\frac{9}{16}$ in. deep. Hæmorrhage was inconsiderable, but the lateral ventricle was opened into. To prevent blood passing from the lateral to the fourth ventricle a strip of iodoform gauze was packed loosely into the ventricle for drainage purposes, and was withdrawn at the end of 48 hours. Healing took place by first intention; six months later the patient's condition was satisfactory, but a later report is promised.

**Pulsating Tumour of Temporal Region.**—Pearson[9] reports a case of successful operation for pulsating tumour of the temporal region of 18 years standing. The man, aged 45, fell from a horse 18 years ago, injuring his head. Pain in the left temporal region ensued a few months later and gradually got worse; he also complained of dizziness and a sensation of fulness in the head on stooping. Still later, a throbbing swelling appeared in the temporal region. It was diagnosed that this swelling was aneurismal, and probably communicated with a vascular swelling inside the skull. An operation was performed in October, 1894, the superficial temporal being first ligatured, and then the large vascular mass removed by means of Paquelin's cautery knife. Hæmorrhage was very free, but was arrested by the cautery. The patient was free of all head symptoms a considerable time after the operation, hence it was concluded that there was no intracranial complication.

[1] Jour. of Nervous and Mental Diseases, Dec., 1896. [2] Brain, 1896. [3] Beiträge zur Klinschen Chirurgie, Bd. XV. [4] Med. Chronicle, Sept., 1896. [5] Glasgow Medical Jour., Nov., 1896. [6] Ep. B. M. J., Jan. 2, 1897. [7] Amer. Jour. Med. Sc., Sept. 1896. [8] Amer. Jour. Med. Sc., Nov, 1896. [9] Dublin Jour. Med. Sc., Dec., 1896.

